# A New Topical Eye Drop Containing LyeTxI-b, A Synthetic Peptide Designed from A *Lycosa erithrognata* Venom Toxin, Was Effective to Treat Resistant Bacterial Keratitis

**DOI:** 10.3390/toxins11040203

**Published:** 2019-04-04

**Authors:** Carolina Nunes da Silva, Flavia Rodrigues da Silva, Lays Fernanda Nunes Dourado, Pablo Victor Mendes dos Reis, Rummenigge Oliveira Silva, Bruna Lopes da Costa, Paula Santos Nunes, Flávio Almeida Amaral, Vera Lúcia dos Santos, Maria Elena de Lima, Armando da Silva Cunha Júnior

**Affiliations:** 1 Faculdade de Farmácia, Universidade Federal de Minas Gerais, Belo Horizonte 31270-901 MG, Brazil; laysndourado@gmail.com (L.F.N.D.); rummeniggeita@gmail.com (R.O.S.); brunalc1991@gmail.com (B.L.d.C.); armando@farmacia.ufmg.br (A.d.S.C.J.); 2 Programa de Pós-Graduação em Ciências Aplicadas à Saúde-PPGCAS, Universidade Federal de Sergipe, Lagarto 49400-000 SE, Brazil; dra.flaviarodrigues@hotmail.com (F.R.d.S.); paulanunes_se@yahoo.com.br (P.S.N.); 3 Departamento de Bioquímica e Imunologia, Instituto de Ciências Biológicas, Universidade Federal de Minas Gerais, Belo Horizonte 31270-901 MG, Brazil; reispvm@gmail.com (P.V.M.d.R.); dr.famaral@gmail.com (F.A.A.); 4 Departamento de Microbiologia, Instituto de Ciências Biológicas, Universidade Federal de Minas Gerais, Belo Horizonte 31270-901 MG, Brazil; verabio@gmail.com; 5 Programa de Pós-graduação em Ciências da Saúde, Biomedicina e Medicina, Ensino e Pesquisa da Santa Casa de Belo Horizonte, Grupo Santa Casa de Belo Horizonte, Belo Horizonte 30150-250, MG, Brazil; mariaelena@santacasabh.org.br

**Keywords:** LyeTxI-b, *Staphylococcus aureus*, keratitis

## Abstract

Bacterial keratitis is an ocular infection that can lead to severe visual disability. *Staphylococcus aureus* is a major pathogen of the eye. We recently demonstrated the strong antimicrobial activity of LyeTxI-b, a synthetic peptide derived from a *Lycosa erithrognatha* toxin. Herein, we evaluated a topical formulation (eye drops) containing LyeTxI-b to treat resistant bacterial keratitis. Keratitis was induced with intrastromal injection of 4 × 10^5^ cells (4 µL) in New Zealand female white rabbits. Minimum inhibitory concentration (MIC) and biofilm viability were determined. LyeTxI-b ocular toxicity was evaluated through chorioallantoic membrane and Draize tests. One drop of the formulation (LyeTxI-b 28.9 µmol/L +0.5% CMC in 0.9% NaCl) was instilled into each eye four times a day, for a week. Slit-lamp biomicroscopy analysis, corneal histopathological studies and cellular infiltrate quantification through myeloperoxidase (MPO) and N-acetylglucosaminidase (NAG) detection were performed. LyeTxI-b was very effective in the treatment of keratitis, with no signs of ocular toxicity. Planktonic bacteria MIC was 3.6 µmol/L and LyeTxI-b treatment reduced biofilm viability in 90%. LyeTxI-b eliminated bacteria and reduced inflammatory cellular activity in the eyes. Healthy and treated animals showed similar NAG and MPO levels. LyeTxI-b is a potent new drug to treat resistant bacterial keratitis, showing effective antimicrobial and anti-inflammatory activity.

## 1. Introduction

The eye is relatively resistant to microorganisms and most pathogens cannot cross the intact cornea, because the eye provides a diverse collection of antimicrobial factors, especially in the tear film, which protects the cornea from infection [1]. However, if there is any structural damage or failure in the defense mechanisms that maintain its entire surface, opportunistic infections can easily develop, resulting in microbial keratitis [1,2,3]. 

Microbial keratitis is characterized by a defect in the corneal epithelium with stromal inflammation caused by microorganisms. The onset of symptoms is acute, causing pain and risking loss or reduction of vision, which requires rapid diagnosis and treatment. Ocular trauma, incorrect use of contact lenses, ocular surface diseases, and ocular surgeries are some factors that can trigger the disease [4,5]. Bacterial keratitis is considered one of the most serious ocular conditions in the world and may cause partial or total loss of visual acuity [4,5,6]. Bacterial infections are still predominant and are found in 80% of patients with ulcerative keratitis [3]. *Staphylococcus aureus* is a major pathogen of the eye, being a natural inhabitant of the ocular surface, skin, nostrils, and environment [7]. *S. aureus* is able to infect the tear duct, eyelid, conjunctiva, cornea, and the anterior, posterior and vitreous chambers. 

Although the incidence of *S. aureus* eye infections varies worldwide, the growing trend of resistance to antibiotics makes this condition an important global healthcare issue. The therapeutic arsenal against bacterial infections is rapidly shrinking and the development of novel antibiotics overcoming antimicrobial resistance is therefore urgent and in great demand. Antimicrobial peptides (AMPs), which act as protective agents against microbes, have been recognized as powerful novel weapons against pathogenic organisms that are resistant to customary antibiotics, because of their unique action mechanisms and broad-spectrum activities [8,9,10]. Today, new drugs are necessary to combat the emergence of antibiotic-resistant germs [11].

Spider venoms contain diverse bioactive peptides and toxins, which have attracted great attention, making them a valuable resource for drug discovery, and excellent candidates for developing novel antibiotics against drug-resistant bacteria [10,12].

LyeTxI is an antimicrobial peptide purified from *Lycosa erythrognatha* spider venom. This peptide was characterized and chemically synthesized [12]. LyeTxI was able to inhibit the proliferation of periodontal bacteria and epithelial cells, and was not cytotoxic to osteoblasts [13]. It can be used to prevent biofilm development and was active against periodontopathic bacteria, showing rapid bactericidal effect [14]. 

Considering that LyeTxI presents great antimicrobial potential, its structure was used as a template to develop new drugs against bacteria. Its derivative synthetic peptide LyeTxI-b has a well-defined helical segment and is 10-fold more effective against gram-positive and gram-negative planktonic bacteria if compared with the natural peptide LyeTxI. The derivative LyeTxI-b differs from LyeTxI only by a lacking histidine residue. It was very effective in treating in vivo septic arthritis in a mouse model [15]. In addition, this synthetic peptide was not toxic to rabbits’ eyes after intravitreal injection and was able to prevent neovascularization in the chorioallantoic membrane, at Bevacizumab levels [16]. Thus, the main goal of this work was to check the effectiveness of LyeTxI-b to the treat in vivo resistant *S. aureus* keratitis by topical application.

## 2. Results

### 2.1. Antimicrobial Activity in Planktonic and Biofilm Culture

To evaluate the minimum inhibitory concentration (MIC) and to determine the minimum biofilm eradication concentrations (MBEC), the formulation (LyeTxI-b +0.5% CMC in 0.9% NaCl) was serially diluted, from 927.7 to 1.8 μmol/L, as previously described in [15] and incubated for MIC test with 5 × 10^4^ CFU/well for 24 h at 37 °C. For MBEC test, the bacterial suspensions had this same densityfor 24 h at 37 °C. Positive (without LyeTxI-b) and negative (without bacteria) controls were submitted to the same procedures as described in methods.

**Figure 1:**
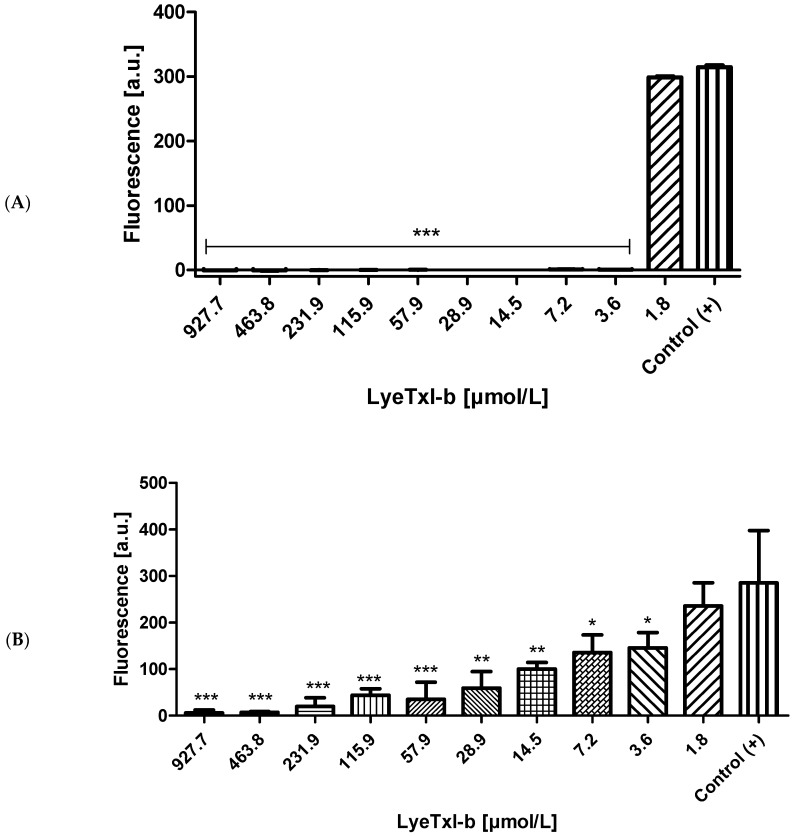
LyeTxI-b action on *S. aureus* planktonic bacteria (**A**) and biofilm (**B**) with different concentrations of the peptide: 1.8–927.7 μmol/L. The viable mass was fluorometrically measured with resazurin (λex 570 nm and λem 590 nm). a.u., arbitrary units. Data are expressed as the mean ± SD. Asterisk indicates LyeTxI-b vs. Control (+) one-way ANOVA plus Bonferroni post-test * *p* < 0.05; ** *p* < 0.01; *** *p* < 0.001.

LyeTxI-b showed potent antimicrobial activity against gram-positive planktonic cells and *S. aureus* established biofilms, reducing their viability under aerobic conditions (Figure 1A,B). The MIC values were 3.6 μmol/L and MBEC was 57.9 μmol/L for LyeTxI-b formulation. 

### 2.2. Ocular Toxicity of the LyeTxI-b by HET-CAM Assay

In the HET-CAM test, embryonated eggs were used to test the toxicity of LyeTxI-b formulation. In vitro tests indicated that the MBEC was 57.9 μmol/L. Based on these data, we performed a toxicity curve. For this, we chose two points below and one point above the MBEC, thus the four test concentrations of LyeTxI-b were: 14.5 μmol /L, 28.9 μmol/L, 57.9 μmol/L and 115.9 μmol/L. Note that 0.5% CMC in 0.9% NaCl was used as the negative control and 0.1 M sodium hydroxide as the positive control. The ocular irritation index (OII) was calculated according to the expression described in methods.

**Figure 2:**
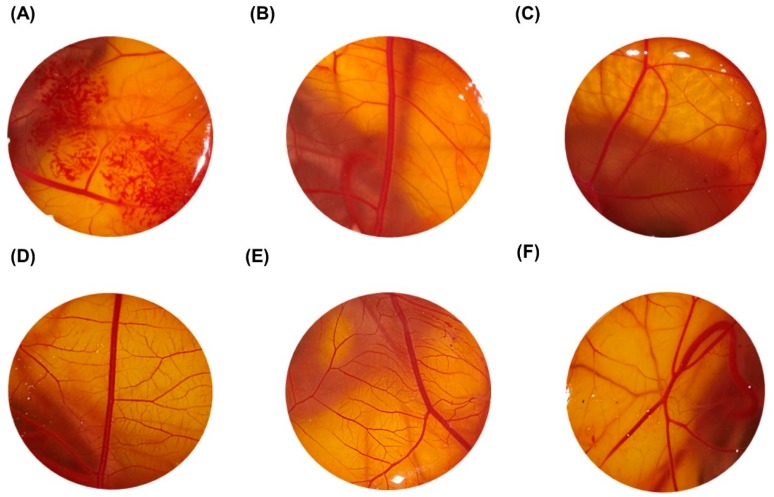
Images of HET-CAM after 5 min exposure to: (**A**) 0.1M NaOH (positive control); (**B**) 0.5% CMC in 0.9% NaCl (negative control); and treatment with LyeTxI-b eye drop at the following concentrations: (**C**) 14.5 μmol/L; (**D**) 28.9 μmol/L; (**E**) 57.9 μmol/L; and (**F**) 115.9 μmol/L.

The results showed that, in the positive control, initial injuries were observed in the first 30 s, as hemorrhage and rosette-like coagulation, which further increased within 5 min (Figure 2). The average cumulative score of the positive control of 0.1 M NaOH was 21.11 ± 0.32 (Figure 2A and Table 1). In the negative control, no modifications were observed after 5 min, and the OII was ≤0.9 ± 0.0 (Figure 2B and Table 1). LyeTxI-b formulation, at the four tested concentrations, showed no signs of vascular response (Figure 2C–F), and the average cumulative scores were ≤0.9 ± 0.0, which categorized this formulation as non-irritant when applied on CAM surface (Table 1).

### 2.3. LyeTxI-b Was Not Irritant for Topical Administration

The total scores for all concentrations of LyeTxI-b were validated between 0 and 3 in long-term eye irritation test. The results show that the formulation with LyeTxI-b did not stimulate irritation on rabbit eye tissues. The corneal and iris scores were zero (Table 2). Although conjunctival hyperemia was observed in the group treated with 115.9 μmol/L, no acute reactions by the rabbits and no prolonged or delayed toxicity were observed. 

### 2.4. LyeTxI-b Reduces Bacterial Growth in Ocular Keratitis

Considering our positive results regarding LyeTxI-b activity in the MIC trial, its ability to reduce biofilm viability and its behavior on ocular tolerance test, we evaluated the in vivo efficacy of this peptide on *S. aureus*-induced keratitis model in rabbits. For these tests, the dose selected was 28.9 µmol/L, which was eight times higher than the MIC and was able to reduce around 50% of the biofilm formation.

**Figure 3:**
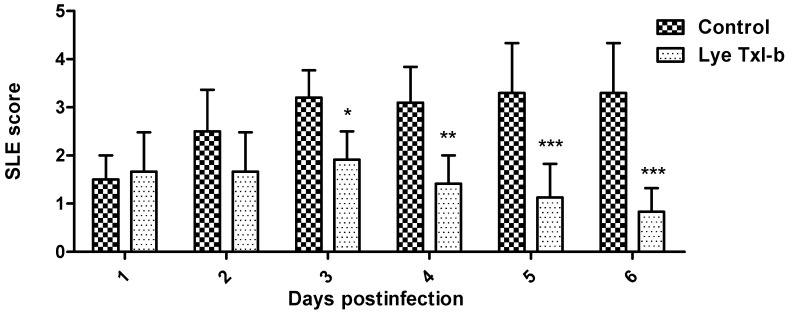
Slit lamp examination (SLE) of ocular disease after infection with *S. aureus*. The ocular disease was graded, and mean SLE scores were calculated by summing the scores for each group (*n* = 6) of rabbits divided by the total number of rabbits graded at each time point. Control: vehicle; Lye TxI-b: LyeTxI-b eye drop. Data are expressed as the mean ± SD. Asterisk indicates LyeTxI-b vs. Control (+) two-way ANOVA plus Bonferroni post-test * *p* < 0.05; ** *p* < 0.01; *** *p* < 0.001.

**Figure 4:**
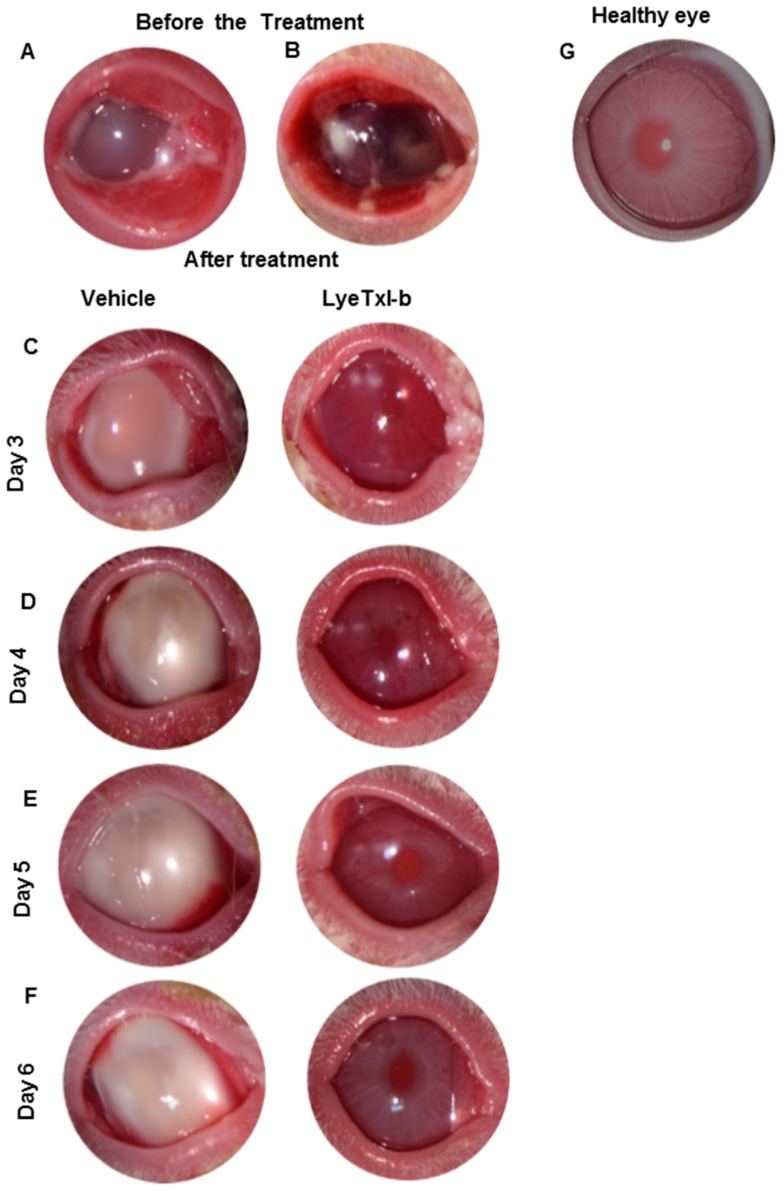
Photomicrographs of the ocular disease in rabbits at post infection (PI) with *S. aureus*. (**A**) Eye control: infected with *S. aureus* before receiving the vehicle. (**B**) Eye infected with *S. aureus* before receiving the LyeTxI-b eye drop. (**C**) Vehicle: three days PI with *S. aureus* treatment with vehicle; and LyeTxI-b: three days PI with *S. aureus* treatment with LyeTxI-b. (**D**) Vehicle: four days PI with *S. aureus* treatment with vehicle; and LyeTxI-b: four days PI with *S. aureus* treatment with LyeTxI-b. (**E**) Vehicle: five days PI with *S. aureus* treatment with vehicle; and LyeTxI-b: five days PI with *S. aureus* treatment with LyeTxI-b. (**F**) Vehicle: six days PI with *S. aureus* treatment with vehicle; and LyeTxI-b: six days PI with *S. aureus* treatment with LyeTxI-b. (**G**) Healthy eye.

The results show that LyeTxI-b was able to significantly reduce bacterial growth. Rabbit eyes were examined for changes through slit lamp examination (SLE) every 24 h. The ocular parameters (according to methods, Table 3) was graded on a scale of 0 (none) to 4 (severe). Figure 3 shows gross pathological signs of infection and significant increases in SLE scores in the control, and these signs significantly reduced after treatment with LyeTxI-b. Corneas infected with bacteria showed a visible increase in haze and clinical deterioration if compared with treated corneas (Figure 4). 

The observed pathological changes in rabbits’ eyes included severe iritis, corneal infiltrates and erosions. At Post-Infection (PI) Day 1 with treatments, some corneal opacity was observed in both control and LyeTxI-b eye-drop-treated (+1.5 to +2.0) rabbits (Figure 3). Dense corneal opacity was observed in infected rabbits’ eyes after PI Day 2 with vehicle (+2.5 to +3) (Figure 3). The animals that received the eye drop treatment showed inhibited disease progression and a significant reduction in corneal opacity density after PI Day 4 (+1.0 to +1.5) (Figure 3 and Figure 4). The disease did not progress beyond (+2.5 to +3.5) after PI Day 6 in controls (Figure 3 and Figure 4). In addition, dense opacities that covered the entire corneal surface and corneal erosions were observed in control on PI Day 6 (Figure 3 and Figure 4). The same was not observed in the animals that received the eye drop with LyeTxI-b (Figure 3 and Figure 4).

**Figure 5:**
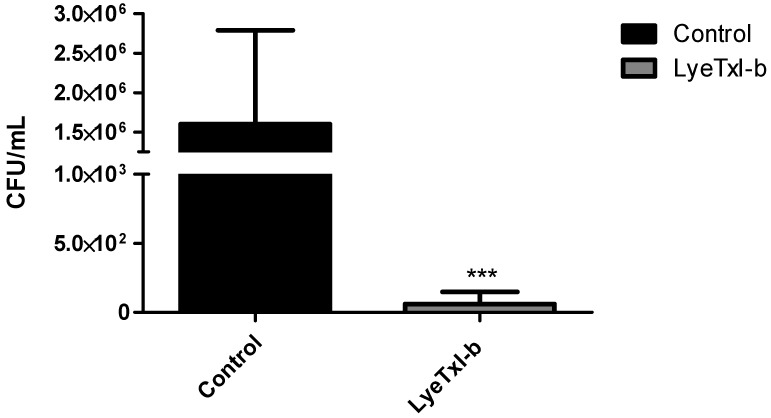
Quantification of viable *S. aureus* in infected rabbit eyes. CFU per infected rabbits eye (Control and LyeTxI-b (28.9 µmol/L), *n* = 6/group) after topical inoculation of *S. aureus* (1 × 10^8^ CFU). Data are expressed as the mean ± SD. Asterisk indicates Control vs. LyeTxI-b treatment unpaired *t*-Student test, *** *p* < 0.001.

To further characterize the course of infection and the treatment with LyeTxI-b eye drop, we quantified the number of viable *S. aureus* (CFU) recovered from infected rabbits’ eyes on PI Day 6. The number of CFU recovered from infected eyes without treatment (Control) was significantly higher when compared to treated eyes (LyeTxI-b) (Figure 5).

### 2.5. Eyes after LyeTxI-b Treatment Show Tissue Repair

**Figure 6:**
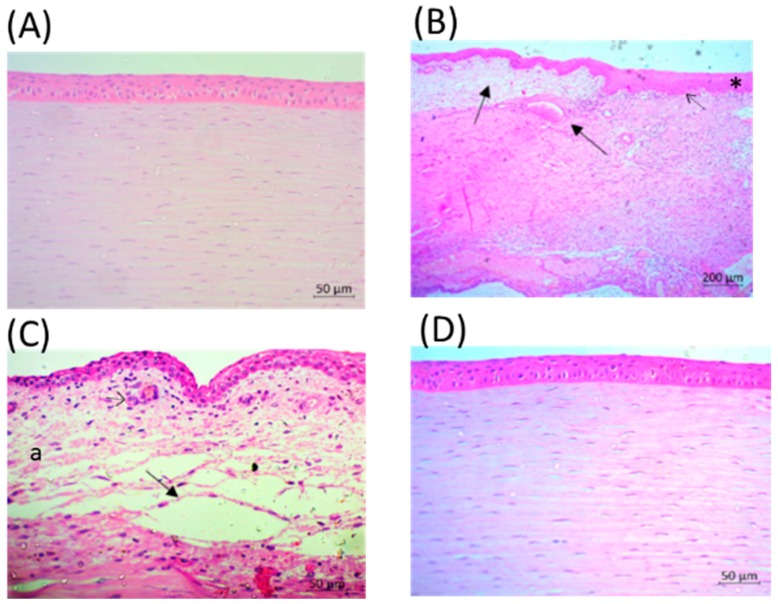
Histopathology of rabbit corneas. (**A**) Healthy cornea. (**B**,**C**) Cross section of a cornea infected with *S. aureus* on PI Day 6. (**B**) Corneal stroma showing vascular congestion, severe edema (arrows), an increase in the corneal epithelium (asterisk) and destruction of the Bowman’s membrane (dotted arrow). (**C**) Severe corneal edema (arrow), stroma infiltrate with polymorphonuclear cells (dotted arrow) and serous exudate accumulation between the collagenous fibers (a). (**D**) Cornea infected with *S. aureus* treated with LyeTx1-b on PI Day 6. (**A**,**C**,**D**) 20× objective; and (**B**) 5× objective.

Histopathologic analysis of the infected eyes demonstrated congestion of the vascularized tissue of the anterior chamber (Figure 6B,C) and severe edema (Figure 6C). In addition, there was an increase in the corneal epithelium, stroma infiltrate with polymorphonuclear cells, erythrocyte diapedesis, serous exudate accumulation between the collagenous fibers, and destruction of the Bowman’s membrane (Figure 6B,C) on PI Day 6.

In contrast, eyes treated with LyeTxI-b eye drops showed intact anterior epithelium and Bowman’s membrane demonstrated few polymorphonuclear infiltrations of the stroma and anterior limiting membrane, minor edema and minor change of collagenous fibers in the stroma (Figure 6D).

### 2.6. PMN Infiltration was Lower After Treatment with LyeTxI-b Eye Drops

**Figure 7:**
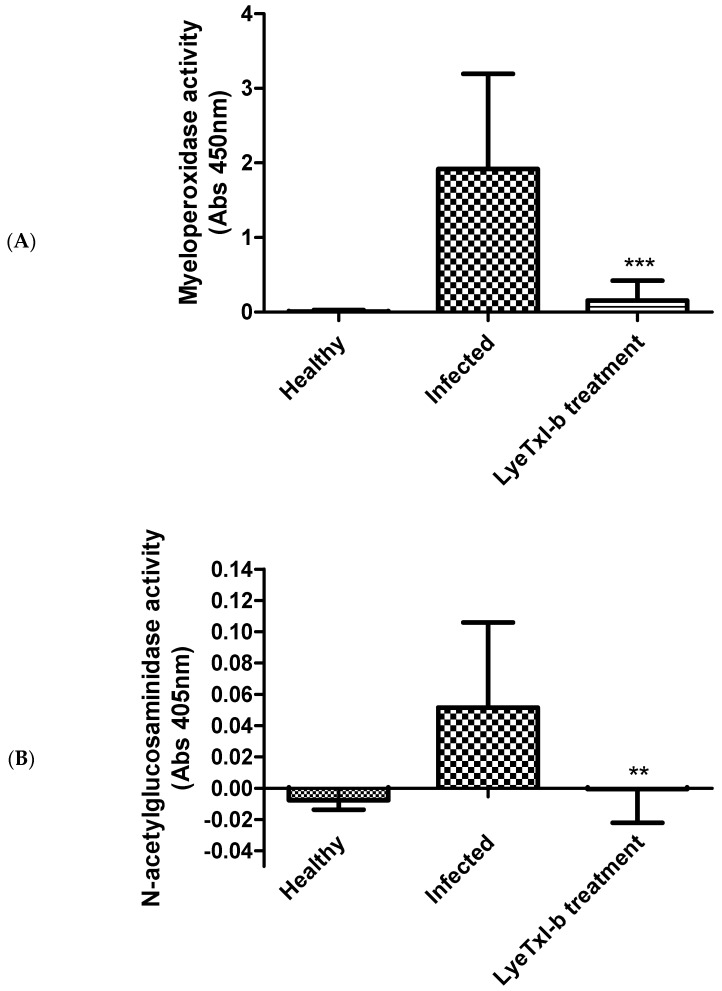
Evaluation of PMN infiltrates by the activity of Myeloperoxidase (MPO) and N-acetylglucosaminidase (NAG) enzymes in the cornea of infected animals: (**A**) MPO activity; and (**B**) NAG activity. *n* = 6, results expressed as mean ± SD. Asterisk indicates LyeTxI-b (28.9 µmol/L) treatment vs. Infected one-way ANOVA plus Bonferroni post-test ** *p* < 0.01; *** *p* < 0.001.

We assessed the leukocyte recruitment by measuring the activity of myeloperoxidase (MPO) and N-acetylglucosaminidase (NAG) in the rabbits’ eye. The results show that, after treatment with LyeTxI-b, there was a significant reduction in the activity of MPO and NAG (*p* < 0.001), being close to the values of healthy animals (Figure 7). 

## 3. Discussion

The main finding of this study was the effectiveness of LyeTxI-b eye drops in the treatment of resistant *S. aureus* keratitis in rabbits and its ability to reduce the inflammatory process in consequence of this infection, which shows LyeTxI-b as a promising antimicrobial agent, corroborating the previous results [15]. Nowadays, there has been an alarming evolution of antimicrobial resistance. Antibiotics are powerful drugs that disrupt or change the composition of the infectious agent and are used to combat severe diseases. As with any powerful medication, the appropriate use of such agents has a highly beneficial effect. However, when improperly used, it leads to bacterial adaptation or mutations and, in turn, to new strains that are resistant to the current antibiotic regiment. In the United States, antibiotic resistance kills around 23,000 patients a year [17]. 

Novel drugs for gram-positive multidrug-resistant bacteria, such as *Staphylococcus aureus*, may contribute in the reduction of bacterial spread and the rate of treatment failure. *S. aureus* is able to form biofilms when colonizing tissues by an aggregation of bacterial cells immobilized in an adhesive extracellular polymeric matrix [18,19]. This hampers its eradication, mainly due to the barrier preventing drug entry or host clearance mechanisms [20,21,22]. Furthermore, an excessive and harmful inflammatory response can be triggered, since the toxins released by the bacteria contribute to the recruitment of immune cells.

Several organisms have developed an arsenal of host-defense molecules, aiming at controlling microbial proliferation and other biological or physical insults, including antimicrobial peptides (AMPs). Spider venoms represent a rich source of AMPs against infectious pathogens. An example is LyeTxI, a peptide isolated from *Lycosa erithrognata* venom that is active against fungi (*Candida krusei* and *Cryptococcus neoformans*) and bacteria (*Escherichia coli* and *S. aureus*) and was able to alter the permeabilization of α-phosphatidylcholine-liposomes in a dose-dependent manner [12]. Its derivative, LyeTxI-b peptide, if compared with LyeTxI, has a deletion of a His residue and is acetylated in its N-terminal portion. These modifications altered its structure and improved its antimicrobial activity in vitro and in vivo. In addition, LyeTxI-b produces lethal pores and membrane-damaging effects on bacteria, besides being effective to treat septic arthritis in mouse [15]. 

To check the efficiency of this peptide as an antibacterial agent in the ophthalmic system, we formulated an eye drop containing LyeTxI-b using carboxymethylcellulose (0.5%) polymer, which was chosen for its physical properties, such as viscosity and mucoadhesiveness, which contribute to its prolonged retention time in the ocular surface [23].

In this work, LyeTxI-b eye drops showed potent antimicrobial inhibition activity against planktonic *S. aureus* (Figure 1A) and were able to eradicate the *S. aureus* biofilm (Figure 1B) only at the highest concentrations tested (463.8 and 927.7 µmol/L). However, at concentrations above 3.6 μmol/L, the peptide potently reduced biofilm when compared with the control group. 

It has been reported that, in a biofilm environment, bacteria could increase the production and excretion of molecules, such as polysaccharides and DNA, in the biofilm matrix, and these molecules, which are negatively charged, would interact with cationic peptides, preventing their binding to the bacterial membrane [24,25]. This could explain the reduction of LyeTxI-b activity in biofilms. 

Considering the positive results regarding LyeTxI-b activity in the in vitro assay, we evaluated its possible toxic effects as a consequence of the instillation, aimed at treating keratitis infection. Our results report, for the first time, that LyeTxI-b formulation, at all tested concentrations, was non-irritant when applied on CAM (Figure 2). In contrast, the positive control (NaOH) was adequate for quality control because it was severely irritant, showing a high score in OII (Table 1). The HET-CAM test, as a model for the study of the precision and safety, can provide information about the conjunctiva ocular effects that formulations may develop. The chorioallantoic membrane is analogous to human retina and its vasculature. Therefore, the irritation with a risk of vascular damage such as hemorrhage, lysis and coagulation can be evaluated for ocular formulations [26,27].

Despite the non-irritancy of LyeTxI-b formulation in the HET-CAM test, Draize test was also performed for the full understanding of ocular tolerance. No signs of ocular inflammation, corneal opacity, conjunctival congestion, swelling or discharge were observed for the doses of 14.5, 28.9 and 57.9 μmol/L of LyeTxI-b during the time of analysis (seven days). However, conjunctival congestion was observed at 115.9 μmol/L of the peptide and this reaction disappeared after one day (Table 2).

Convinced that the peptide was safe for ocular use, we induced the bacterial keratitis in rabbits and evaluated the efficiency of the LyeTxI-b formulation to treat the infection. The results obtained in this work (Figure 3 and Figure 4) turn clear the potential use of LyeTxI-b to treat bacterial keratitis. LyeTxI-b, at a low dose (28.9 µmol/mL), could eliminate penicillin-, erythromycin- and ampicillin-resistant *S. aureus* if compared to ciprofloxacin (1.5 mMol/mL), although, at first, they present different mechanisms of action. O’Callaghan [1] showed that ciprofloxacin was more effective than vancomycin or cefazolin in the early stages of cornea infection with methicillin-resistant *S. aureus*. The concentration of LyeTxI-b eye drop was near to one hundred times lower if compared to ciprofloxacin, which indicates its higher potency. A similar effect was observed when LyeTxI-b was used for the treatment of septic arthritis: the peptide decreased the bacterial load to the same level as clindamycin, but LyeTxI-b concentration was two hundred times lower [15].

Several pathological changes could be observed in infected control eyes, but the same was not observed in the eyes treated with LyeTxI-b eye drops. Keratitis resulting from intrastromal injection is characterized by bacterial replication and severe ocular changes, including edema, corneal epithelial cell destruction, iritis, as well as the migration of polymorphonuclear neutrophils (PMNs) from the eyelid to the tear film [28,29]. All these changes were observed in the animals that received vehicle (Control). Gross signs of infection appeared within 24 h in the infected rabbit eye and progressed in control group beyond +2.5 to +3.5 SLE score. In contrast, the formulation with LyeTxI-b reduced the disease progression almost reaching the morphology of healthy eyes. 

The CFU results highlight the potential of this peptide to treat this disease, as shown in Figure 5. Corroborating these results, the histopathological analysis evidenced the alterations caused by *S. aureus* cornea infection and showed significant improvement after treatment with LyeTxI-b eye drops, including the reduction of cellular infiltrate (Figure 6).

Myeloperoxidase enzyme (MPO) is mainly found in cytoplasmic granules of neutrophils, but also in monocytes. MPO is a protein of the heme group secreted by activated leukocytes. It promotes the conversion of hydrogen peroxide to hypochlorous acid and halides in hypoallergenic acids, leading to the formation of highly reactive intermediates, which in turn contributes to lipid peroxidation [30,31]. NAG, on the other hand, is a lysosomal enzyme highly present in activated macrophages. Together, MPO and NAG serve as good markers of neutrophil and macrophage infiltration in tissues and inflammation, respectively [32]. Our study shows that LyeTxI-b eye drops were able to significantly reduce neutrophil and macrophage activity after topical keratitis induction (Figure 7). 

LyeTxI-b eye drops were safe for ocular use and were able to treat topical and resistant bacterial keratitis in rabbits. In addition, this formulation was able to reduce the inflammatory process triggered by the disease, since there was a significant reduction in the cellular infiltrate or a reduction in the activity of these cells. In conclusion, our results show that LyeTxI-b is an excellent candidate as an alternative drug to treat keratitis.

## 4. Materials and Methods

### 4.1. Materials

The following were used: Mueller–Hinton (MH) broth (Himedia, Mumbai, Índia), brain heart infusion (BHI) (Kasvi, São José do Pinhais, Brazil), mannitol salt agar (Kasvi, São José do Pinhais, Brazil), resazurin, carboxymethylcellulose (CMC) and Dimethyl sulfoxide (DMSO) were purchased from Sigma-Aldrich (Darmstadt, Germany); Ketamin^®^ (Cristália, São Paulo, Brazil); xylazine hydrochloride (Copanize^®^, Schering-Plough Coopers, São Paulo, Brazil); proxymetacaine hydrochloride (Anestalcon; Alcon, São Paulo, Brazil); 3,3′,5,5′-Tetramethylbenzidine (TMB, Sigma-aldrich, Germany); and 4-Nitrophenyl N-acetyl-β-d-glucosaminide (NAG, Sigma-aldrich, Darmstadt, Germany).

The peptide LyeTxI-b (CH3CO-IWLTALKFLGKNLGKLAKQQLAKL-NH2) was synthesized by GenOne Biotechnologies (Rio de Janeiro, Brazil). 

### 4.2. Methods

#### 4.2.1. In Vitro Antimicrobial Test

A strain of *Staphylococcus aureus* resistant to penicillin, erythromycin and ampicillin was isolated from ocular samples of a 22-year-old female patient, in a private clinical analysis laboratory in Belo Horizonte-MG, Brazil. This strain was employed in both planktonic and biofilm forms in this study. BHI broth was used to culture planktonic in aerobic conditions. The MIC using the microdilution method was performed. Samples containing the formulation (LyeTxI-b +0.5% CMC in 0.9% NaCl) were serially diluted from 927.7 to 1.8 μmol/L in MH broth and incubated with 5 × 10^4^ CFU/well for 24 h at 37 °C. The lowest concentration of the peptide formulation that prevented the visible growth of the microorganism was defined as the MIC. 

The determination of minimum biofilm eradication concentrations (MBEC)was performed as previously described [33], with modifications. Bacterial suspensions (10 µL), with the same densityof MIC, were added to 96-well plates with MH medium supplemented with glucose 1% and incubated on a horizontal shaking plate at 37 °C, for 24 h, for biofilm formation. The growth medium was discarded and biofilms were washed with sterile saline. Formulations of LyeTxI-b were added at decreasing concentrations (927.7–1.8 μmol/L). After incubation at 37 °C for 24 h, *S. aureus* biofilms were resuspended, resazurin solution (0.1 g/L) was added and the plates were incubated at 37 °C for 20 min in the absence of light. Then, the plates were read in VarioskanTM (λ ex 570 nm e λ em 590 nm) and MBEC was determined as the lowest concentration of peptide formulation that prevented biofilm formation. Resazurin is an indicator of oxidation-reduction used for the evaluation of cell growth [34]. It is a non-fluorescent blue and non-toxic dye. When reduced to resorufin by oxidoreductases within viable cells, it becomes pink and fluorescent. The level of reduction can be quantified by spectrophotometer.

Positive and negative controls were submitted to the same procedures. In the positive control, there was no addition of any peptide or inhibitory drug, just the formulation (0.5% CMC in 0.9% NaCl). The negative control did not have any bacteria and the value was normalized to zero in all analyses. MIC and MBEC assays were performed in triplicate. 

#### 4.2.2. Evaluation of LyeTxI-b Toxicity by Chorioallantoic Membrane Test (HET-CAM)

HET-CAM is described in [35] and adapted in [36,37]. Ten fertilized chicken eggs were selected for each concentration tested. The eggs were incubated at 37 ± 1 °C and 60 ± 1% relative humidity for 10 days. On the tenth day, the eggshell was opened and the inner membrane of the egg was carefully removed to avoid any damage to the thin vessels of the chorioallantoic membrane. The concentrations of 14.5, 28.9, 57.9 and 115.9 μmol/L of LyeTxI-b +0.5% CMC in 0.9% NaCl (300 μL) were applied to CAM. Note that 0.5% CMC in 0.9% NaCl was used as negative control and 0.1M sodium hydroxide as positive control. The intensity of the reactions was semi-quantitatively evaluated, on a scale from 0 (no reaction) to 3 (strong reaction). The appearance and intensity of all reactions, if any, were observed at 0 s, 30 s, 2 min and 5 min. The ocular irritation index (OII) was then calculated by the following expression, where *h* is the time (in seconds) of the onset of hemorrhage, *l* lysis, and *c* coagulation, over a period of 300 s (5 min). The separate ratio was multiplied by a factor indicating the impact on vascular damage by the observed effect. Thus, coagulation has the highest impact indicated by factor 9 [35,36].

(1) $$OII=\frac{\left(301-h\right)}{300}+\frac{\left(301-l\right)}{300}+\frac{\left(301-c\right)\;x\;9}{300}$$

#### 4.2.3. Ocular Tolerability of LyeTxI-b

Sixteen female New Zealand white rabbits were used. Ocular irritation in the rabbits was evaluated according to the Draize test [38,39] at 1 h, 24 h, 48 h, 72 h and 7 days after the instillation with 4 concentrations 14.5, 28.9, 57.9 or 115.9 μmol/L of LyeTxI-b formulated in 0.5% CMC in 0.9% NaCl, *n* = 4. The method provides an overall scoring system for grading the severity of ocular lesions involving the cornea (opacity), iris (inflammation degree), and conjunctiva (congestion, swelling, and discharge). The Draize score was determined by visual assessment of changes in these ocular structures. Maximum mean total scores (MMTS) were calculated and eye irritation was classified.

#### 4.2.4. Induction of Bacterial Keratitis and Treatment with LyeTxI-b

Twelve female New Zealand white rabbits, aged approximately three months and weighing 2 kg were purchased from the Experimental Farm Professor Hélio Barbosa (Igarapé, MG, Brazil). The animals remained in individual cages throughout the period of adaptation (1 week) and experimentation (7 days) at 25 °C and brightness varying according to sunlight. There was no restriction of water or food during the experiment. The animals were divided into two groups: control (received vehicle) and treated (received LyeTxI-b eye drop). 

The study was approved by the Ethics Committee in Experimentation Animal of the Federal University of Minas Gerais (CEUA, Belo Horizonte, Brazil, Protocol No. 298/2017, date of approval: 11 January 2017). All experiments were conducted in accordance with the Association for Research in Vision and Ophthalmology (ARVO).

The rabbits were anesthetized with intramuscular combination injection of ketamine hydrochloride (30 mg/kg) and xylazine hydrochloride (4 mg/kg) and the eyes were topically anesthetized with 0.5% proxymetacaine hydrochloride. Before injection with bacteria, the eyes were wiped with 5% povidone-iodine.

To induce unilateral keratitis, 4 × 10^5^ colony-forming units/mL (4 µL) suspensions of clinical *Staphylococcus aureus* isolates in logarithmic growth phase were injected into the central corneal stroma in the eye. The injection was performed with a 30-gauge needle. Twenty-four hours after bacterial injection, the rabbits were randomly separated in two groups with six animals each and were submitted to the following treatments: (1) Formulation with Lye TxI-b (28.9 µmol/L); and (2) Control (vehicle 0.5% CMC in 0.9% NaCl). The LyeTxI-b eye drops (20 µL) and vehicle were instilled in rabbits’ eyes into a space (fornix) created by gently pulling down the lower lid, every 6 h, for 6 days. Two hours after the last dose of LyeTxI-b or vehicle, the rabbits were euthanized using an overdose of sodium pentobarbital (81 mg/kg). Corneas were carefully and aseptically removed and cut in three parts. One part was cultured to enumerate viable bacteria, the other was used for histopathology and the third part was used for MPO and NAG quantification. The rabbit’s corneas (20 mg) excised were immediately homogenized in phosphate buffered saline (PBS). The material was serially diluted and 0.1 mL aliquots were plated in triplicate on mannitol salt agar for enumeration of *S. aureus*. The specimens were incubated at 35 °C for 24 h.

#### 4.2.5. Slit Lamp Examination (SLE)

The ocular disease was evaluated both macroscopically and microscopically using a slit lamp biomicroscope (Apramed HS5, São Carlos, Brazil) during the experiment, every 24 h by two masked observers. Observations of *S. aureus*-infected rabbits’ eyes were graded with a modification of the scale previously described [40]. The corneal response was graded from 0 to +4 (Table 3).

#### 4.2.6. Histological Analysis

Immediately after sacrifice, one part of the cornea was removed and fixed in Davidson solution [16]. Samples were included in paraffin and 4-μm-thick sections of the sagittal plane, to allow dorsal-to-ventral observation of the cornea and retina, were stained with hematoxylin and eosin and were analyzed in unmyelinated areas under light microscopy (Zeiss^®^, Model Axio Imager M2, San Diego, California, USA). Eyes that received LyeTxI-b eye drops were compared with the control. 

#### 4.2.7. Inflammatory Analysis: MPO and NAG Activity 

Initially, 20 mg of cornea were homogenized in ice-cold Buffer 1 solution (0.1 M NaCl, 0.02 M Na_3_PO_4_ and 0.015 M Na_2_EDTA) and centrifuged at 4 °C (5000 g, 10 min). The supernatant was discarded and the pellet was resuspended in 0.2% NaCl solution and 1.6% NaCl plus 5% glucose. The samples were homogenized and centrifuged at 4 °C (5000 g, 10 min). The supernatant was discarded and the pellet resuspended in buffer 2 (Na_3_PO_4_ and 0.5% HETAB *w*/*v*) solution. For the MPO assay, this homogenate was frozen in liquid nitrogen and unfrozen in water at room temperature for three consecutive times. The samples were then centrifuged at 4 °C (5000 g, 15 min). An aliquot of the supernatant was removed for dilution in buffer 2 and performance of the enzymatic assay. In the microplate, the sample was plated in triplicate. TMB substrate, previously diluted in DMSO, was added. The plate was placed in an oven at 37 °C for 5 min. Then hydrogen peroxide solution (0.002%) was added and the samples were again incubated at 37 °C for 5 min. After the incubation, the reaction was stopped with the addition of sulfuric acid (1M). The absorbance was read at 450 nm. The mean of the values obtained in each triplicate was used to determine the activity of the enzyme. 

For indirect quantification of N-acetylglucosaminidase (NAG) activity in macrophages, 20 mg of cornea tissue were weighed and homogenized with Saline/Triton solution (Saline 0.9% and Triton x-100, 1%) and then centrifuged at 4 °C (1500 g, 10 min). The supernatant was collected and diluted in phosphate-citrate buffer (0.1 M citric acid and 0.1 M Na_2_HPO_4_) to perform the NAG assay. One hundred microliters of each diluted sample were plated in triplicate. The substrate p-nitrophenyl-N-acetyl-β-d-glucosaminide (2.2 mM), diluted in phosphate-citrate buffer, was added. Samples were incubated in an oven at 37 °C for 5 min. After the reaction, 0.2 M of glycine buffer was added to the samples to paralyze the reaction. The absorbance was read at 405 nm. The mean of the values obtained in each triplicate was used to determine the activity of the enzyme.

#### 4.2.8. Statistical Analysis

Statistical analyses were performed using the GraphPad Prism^TM^ software version 5.0 and data were expressed as mean ± standard deviation (SD), followed by single-variance analysis (ANOVA one-way) with Bonferroni post-test. Results were considered significant for values of *p* < 0.05.

## Figures and Tables

**Table 1:** Ocular irritation index (OII) scores for the tested LyeTxI-b eye drops.

Tested Solution/Dispersion	OII ± SEM	Irritancy Classification
0.1 M NaOH (Positive control)	21.11 ± 0.32	SI
0.9% NaCl +CMC 5% (Negative control)	≤0.9 ± 0.0	NI
LyeTxI-b—14.5 μmol/L	≤0.9 ± 0.0	NI
LyeTxI-b—28.9 μmol/L	≤0.9 ± 0.0	NI
LyeTxI-b—57.9 μmol/L	≤0.9 ± 0.0	NI
LyeTxI-b—115.9 μmol/L	≤0.9 ± 0.0	NI

NI, Non-irritant or slightly irritant; SI, Severely irritant. The results are expressed as mean ± SD (*n* = 10).

**Table 2:** Draize test demonstrating the maximum mean total scores of rabbit eyes treated with several concentrations of LyeTxI-b eye drop.

Location	Concentrations (μmol/L)			
	14.5	28.9	57.9	115.9
Cornea opacity	0	0	0	0
Iris inflammation degree	0	0	0	0
Conjunctival congestion	0	0	0	0.6
Conjunctival swelling	0	0	0	0
Conjunctival discharge	0	0	0	0
Total score	0	0	0	0.6

**Table 3:** Grading of observable ocular disease in infected rabbits.

0 = Clear or slight opacity, partially covering pupil. +1 = Slight opacity covering anterior segment. +2 = Moderate to dense opacity, partially or fully covering entire corneal and over pupil. +3 = Dense opacity, covering entire anterior segment. +4 = Corneal erosion or phthisis.
